# Jacob’s Dream

**Published:** 1866-11

**Authors:** 


### JACOB'S DREAM.

In a beautiful vision of the night, which was at the same time a reality, the young wanderer, in a measure an outcast from his father's house, looked up into heaven and saw its happy angels, and voices came to him so sweetly comforting, that he was waked up from his delicious trance, and soliloquized, "Surely, the Lord is in this place, and I knew it not." He knew not the extent of his happiness until it had passed. So we all look back in memory on the scenes of childhood, and feel that they were in days of sunshine, but we "knew it not" at the time; often know it not until years numbered by tens and twenties have passed, and "gray hairs are upon" us.

